# Automated underwater *plectropomus leopardus* phenotype measurement through cylinder

**DOI:** 10.1038/s41598-025-08863-w

**Published:** 2025-07-08

**Authors:** Mengran Liu, Yaocheng Huang, Guojun Xu, Junwei Zhou, Cun Wei, Jingjie Hu, Zhenmin Bao

**Affiliations:** 1 Key Laboratory of Marine Genetics and Breeding, College of Marine Life Sciences/Key Laboratory of Tropical Aquatic Germplasm of Hainan Province, Ocean University of China, Qingdao, China; 2 School of Computer and Artificial Intelligence, WuHan University of Technology, WuHan, Hubei China

**Keywords:** Keypoints detection, Underwater measurement, Binocular visual, Refraction correction, Image processing, Depth estimation, Marine biology, Computer science

## Abstract

Accurate and non-invasive measurement of fish phenotypic characteristics in underwater environments is crucial for advancing aquaculture. Traditional manual methods require significant labor to anesthetize and capture fish, which not only raises ethical concerns but also risks causing injury to the animals. Alternative hardware-based approaches, such as acoustic technology and active structured light techniques, are often costly and may suffer from limited measurement accuracy. In contrast, image-based methods utilizing low-cost binocular cameras present a more affordable solution, although they face challenges such as light refraction between water and the waterproof enclosure, which can cause discrepancies between image coordinates and actual positions. To address these challenges, we have developed a fish keypoint detection dataset and trained both a fish object detection model and a keypoint detection model using the RTMDet and RTMPose architectures to identify keypoints on Plectropomus leopardus. Since the binocular camera must be housed in a waterproof enclosure, we correct for birefringence caused by the water and the enclosure by applying refraction corrections to the detected keypoint coordinates. This ensures that the keypoint coordinates obtained underwater are consistent with those in air, thereby improving the accuracy of subsequent stereo matching. Once the corrected keypoint coordinates are obtained, we apply the least squares method, in conjunction with binocular stereo imaging principles, to perform stereo matching and derive the actual 3D coordinates of the keypoints. We calculate the fish body length by measuring the 3D coordinates of the snout and tail. Our model achieved **98.6%** accuracy in keypoints detection (AP@0.5:0.95). Underwater tests showed an average measurement error of approximately **3.2 mm** (MRPE=3.50%) for fish in a tank, with real-time processing at **28 FPS** on an NVIDIA GTX 1060 GPU. These results confirm that our method effectively detects keypoints on fish bodies and measures their length without physical contact or removal from the tank. By eliminating invasive procedures, our approach not only improves measurement efficiency but also aligns with ethical standards in aquaculture. Compared to existing techniques, our method offers enhanced accuracy (reducing MRPE by 53.8% compared to baseline methods) and practicality, making it a valuable tool for the aquaculture industry.

## Introduction

The global aquaculture industry, valued at $289 billion in 2022^1^, faces mounting pressure to adopt sustainable practices as wild fish stocks decline at an annual rate of **1.2%**^2^. As living standards rise and fish stocks decline, the aquaculture industry has grown significantly, as fish are a vital source of high-quality protein. This sector primarily operates through three methods: industrial farming, pond culture, and cage culture. Monitoring the growth of farmed fish is crucial for effective management, with accurate body length estimation being essential for assessing growth, weight, and biomass^3^.Traditional manual measurement methods, however, incur substantial costs: a typical fish farm spends **15-20%** of labor hours on growth monitoring^4^, with a **12-18%** mortality rate due to handling stress^5^. Regular collection of body length data is necessary for refining feeding strategies, managing stocking densities, and determining optimal harvest timing. However, traditional growth studies often rely on invasive, manual capture and measurement techniques, which are time-consuming, labor-intensive, and can adversely affect fish health. Thus, developing a non-invasive, rapid, and cost-effective system for measuring fish body length has become a key research focus, aimed at improving efficiency and sustainability in aquaculture practices.

The aquaculture industry is increasingly moving toward large-scale, intensive, and intelligent operations, making the monitoring of fish growth a critical aspect of developing advanced aquaculture technologies. Alongside rapid advancements in computer science, digital imaging, and image processing, computer vision has gained prominence in aquaculture research^6^. As a reliable and non-invasive observation method, computer vision enables the identification of farmed fish in surveillance footage and facilitates size measurements through techniques such as foreground segmentation and advanced image processing. This approach is non-intrusive, offering benefits like precision, stability, and the potential for real-time measurement.However, current vision-based systems achieve only **5.5-7.0%** mean relative error in underwater length measurement^7^, insufficient for precision aquaculture requirements.

In conclusion, the rapid advancement of the aquaculture industry and the national strategy for smart agriculture highlight the urgent need for automated fish body length measurement technology using computer vision. This study focuses on *Plectropomus Leopardus*, for which we establish a dataset for keypoint detection and propose an inexpensive and reliable method for measuring fish length that can be directly applied in underwater environments. While effective for this species, further validation is needed to generalize the approach to other aquaculture scenarios. This innovation enables automatic identification, detection, and measurement of *Plectropomus Leopardus*, contributing to the intelligentization of aquaculture equipment for this species. The proposed method reduces labor costs, enhances technological sophistication, and raises industry standards, positioning it as a key direction for future development^8^.

Despite advancements in computer vision, three critical challenges persist in underwater phenotype measurement:

- **Refractive distortion**: Multi-interface refraction (water-glass-air) causes **3.8-12.4% coordinate errors**^9^, which existing single-medium correction methods fail to address.
- **Species adaptability**: Over 90% of public fish datasets^10^ focus on salmonids, lacking annotations for tropical species like *Plectropomus Leopardus*.
- **Hardware dependency**: Commercial systems (e.g., EventMeasure ®) require $15,000+ setups^11^, limiting accessibility for small-scale farms.

The main contributions of this paper are as follows:

1. We establish a fish keypoint detection dataset consisting of 400 annotated fish images and train both a fish object detection model and a keypoint detection model using the RTMDet and RTMPose architectures to identify Plectropomus Leopardus and its keypoints.
2. Our method utilizes affordable hardware, achieving a measurement error of approximately **3.2 mm**, making it suitable for real-world applications in the aquaculture industry.
3. We address image refraction in tank environments by correcting the detected keypoints, thereby improving the accuracy of 3D coordinate measurements and enhancing fish length estimation under these conditions.
4. Extensive experiments are conducted in various settings, particularly in real-world environments, to validate the effectiveness of our method. These tests were performed in diverse conditions, including both land and water tanks, with the tank environment characterized by high turbidity and fish density, closely mimicking actual production scenarios. These experiments demonstrate the feasibility of the algorithm for practical applications.

## Results

### Implementation details

The fish and keypoint detection model was trained on a server with the configuration of an NVIDIA A100-PCIE-40GB. The AdamW optimizer was used, and the hyperparameter settings during training are shown in Table 1.

**Table 1:** Hyperparameter settings during training.

Parameter Name	Value
Input Size	$512\times 512$
Batch Size	16
Initial Learning Rate	0.004
Decay	0.05
Momentum	0.9
Epochs	500

The initial learning rate was set to 0.004. To prevent overfitting, the learning rate was reduced to one-tenth at epoch 250. As the training progressed, the loss gradually decreased and converged to around 0.02. The evaluation metric used to measure the accuracy of the fish keypoint detection is NME, a common metric for keypoint accuracy, which can be applied here. The NME for each image is defined as:

1 $$\begin{aligned} NME(P,{\hat{P}}) = \frac{1}{N} \sum _{i=1}^{N} \frac{\Vert {\textbf{p}}_i - \hat{{\textbf{p}}}_i \Vert _2}{d} \end{aligned}$$

Where ’N’ is the number of keypoints, ’P’ represents the true keypoint coordinates, ${\hat{P}}$ is the predicted keypoint coordinates, and ’d’ is the diagonal length of the face bounding box, which normalizes the error. A decreasing NME value over time during training indicates that the detected keypoints are progressively closer to the true positions.

Thirteen groupers Plectropomus Leopardus of varying sizes (ranging from 8 cm to 10 cm in length) were placed in separate water tanks. A calibrated binocular camera was positioned outside the tank to capture images of the fish through the tank wall. These images were processed using the keypoint detection algorithm based on RTMPose, which operates in a top-down manner. The pixel coordinates of the detected keypoints were then corrected to approximate their positions in air, compensating for refraction effects using the refraction correction algorithm. The corrected pixel coordinates were converted into relative 3D coordinates using the 3D mapping algorithm. Finally, the Euclidean distance between the keypoints at the mouth and tail of each fish was calculated, and the relative error between the system-calculated lengths and the manually measured lengths was statistically analyzed.

### Keypoints detection based on RTMPose

First, the 69 images in the test set of Fish-Keypoints were used to evaluate both the target detection and keypoint detection tasks. Confidence refers to the model’s belief that a target exists within the detection box. For each detection box, it is first filtered using a confidence threshold. If the confidence level of the box exceeds the threshold, the target is considered present (i.e., a positive sample); otherwise, it is considered absent (i.e., a negative sample). A higher confidence threshold results in fewer generated target boxes, but the certainty that a target exists in each remaining box increases.

For quantitative analysis, the model’s performance was evaluated using the commonly used target detection metric AP(average precision). To refine the network’s performance, we calculated AP values under various Intersection over Union (IoU) thresholds. A higher IoU threshold requires stricter alignment to classify a detection as a positive sample. If the model maintains a high AP value at higher IoU thresholds, it indicates better model performance. Similarly, for keypoint detection, AP values were computed under different IoU thresholds.

When the IoU threshold was set to 0.5, the accuracy of target detection was $98.9\%$, and the accuracy of keypoint detection was $98.7\%$. At an IoU of 0.75, both tasks maintained an AP value nearly identical to that at 0.5. However, at an IoU of 0.95, the AP value for both tasks showed some decline, though the keypoint detection task still maintained a high level of performance. The average accuracies across ten IoU thresholds (0.50 : 0.05 : 0.95) were $98.6\%$ for target detection and $99.1\%$ for keypoint detection. The model was capable of achieving up to 60 frames per second (FPS) on the mentioned hardware configuration, balancing both performance and speed. These results demonstrate that the model exhibits good generalization in underwater scenarios.

The detection accuracy of the proposed system was further evaluated under three different water turbidity conditions: clear water, moderate turbidity, and high turbidity. As shown in Table 2, the system performed with high accuracy in clear water, achieving a detection accuracy of 99.2%. Under moderate turbidity, the accuracy slightly decreased to 98.8%, and under high turbidity, the accuracy further declined to 98.2%. These results indicate that while the model excels under clear water conditions, it remains robust even in varying levels of water turbidity, highlighting its effectiveness in real-world underwater environments.

**Table 2:** Detection accuracy under different water turbidity conditions.

Water Turbidity Condition	Detection Accuracy (%)
Clear Water	99.2%
Moderate Turbidity	98.8%
High Turbidity	98.2%

### Fish body length estimation

To validate the accuracy of the proposed contactless method for measuring the body length of Plectropomus Leopardus in water, as well as the feasibility of adaptive measurement and precision of calculation, a series of experiments were conducted. The experiments were performed in three groups with a water depth of 500 mm in the fish tank. The experimental methods were as follows: (1) before refraction correction, (2) after refraction correction, and (3) after both refraction correction and underwater calibration. In each experimental group, Plectropomus Leopardus were randomly selected for measurement, with ten measurements taken per fish. To ensure accuracy, images of fish with different shapes were captured for each measurement by adjusting the camera position horizontally on a tripod or by stirring the position of the fish underwater.

The relative error (RE) is defined as the relative difference between the estimated and true values:

2 $$\begin{aligned} \text {RE} = \frac{|L_{ti} - L_{ai}|}{L_{ai}} \times 100\% \end{aligned}$$

where $L_{ti}$ represents the distance measured by the algorithm, and $L_{ai}$ represents the actual distance measured manually.

The mean relative percentage error (MRPE) was used to evaluate measurement performance:

3 $$\begin{aligned} \text {MRPE} = \frac{1}{n} \sum _{i=1}^{n} \left| \frac{L_{ti} - L_{ai}}{L_{ai}} \right| \times 100\% \end{aligned}$$

Table 3 shows the MRPE for each experimental group, compared to manual measurements (ML), after incorporating refraction correction algorithms and underwater calibration methods. Group 1 does not account for water refraction or use underwater calibration camera parameters. Group 2 only considers water refraction, while Group 3 considers both water refraction and uses underwater calibration camera parameters. The MRPE for fish length in Groups 1, 2, and 3 were 7.80%, 3.66%, and 3.50%, respectively. As the methods improved, the measurement error decreased, indicating that water refraction and underwater calibration parameters should be considered during measurement.

**Table 3:** MRPE (Mean Relative Percentage Error) for three groups.

No.	ML/mm	Group 1	Group 2	Group 3
1	82.8	11.71%	3.62%	4.35%
2	88.2	14.85%	8.05%	6.80%
3	89.4	5.81%	0.34%	4.25%
4	90.7	9.59%	7.94%	1.43%
5	92.4	2.16%	0.43%	2.49%
6	92.5	2.48%	3.14%	3.78%
7	93.4	8.99%	1.50%	1.93%
8	93.5	6.09%	1.39%	0.43%
9	95.5	11.31%	4.92%	1.05%
10	96.1	1.97%	2.71%	4.06%
11	97.1	4.73%	0.21%	0.51%
12	99.5	17.08%	10.25%	9.45%
13	103.6	11.49%	4.05%	4.36%
**MRPE**		7.80%	3.66%	3.50%

**Fig. 1:**
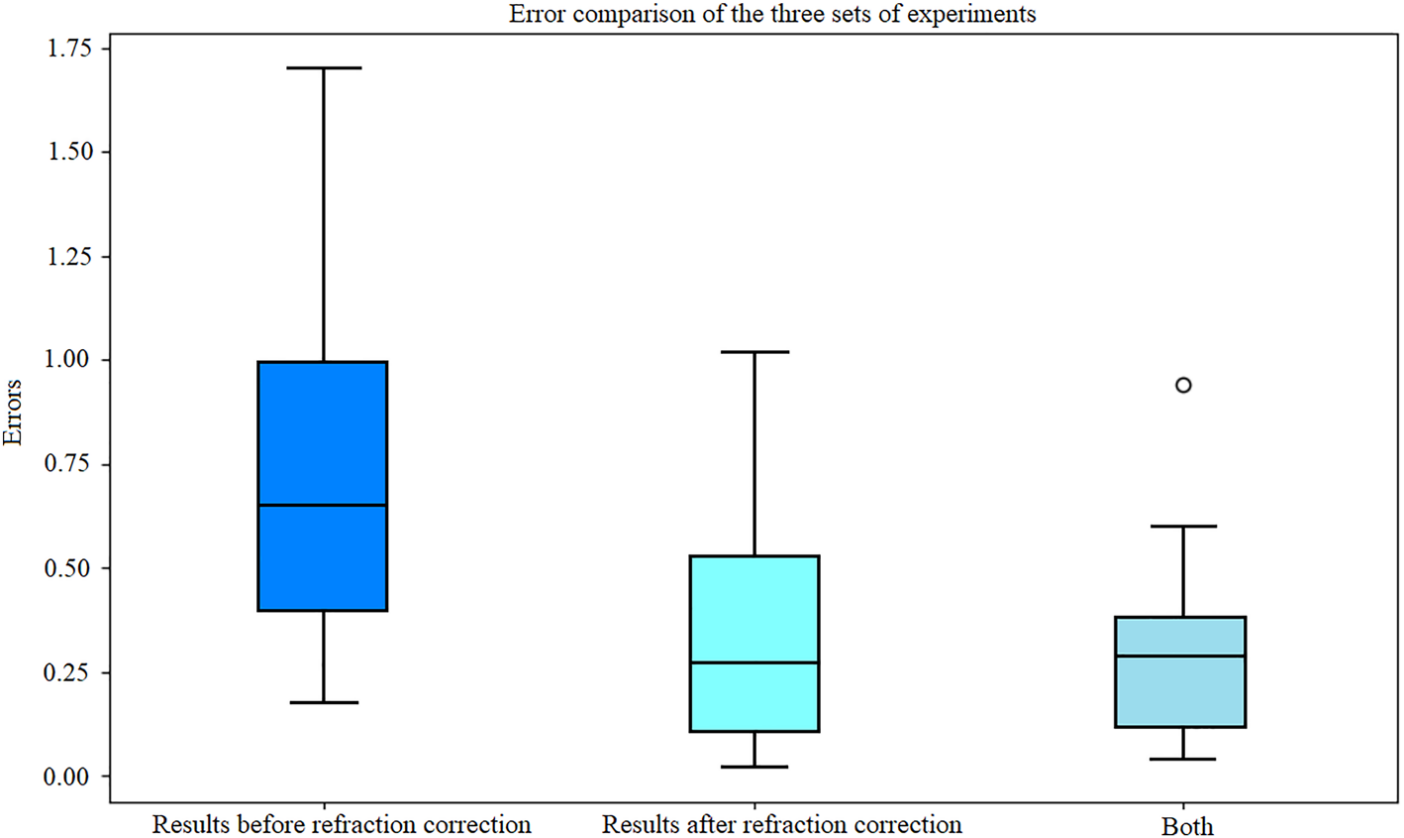
Error comparison of the three sets of experiments.

Figure 1 shows the RE values for the three sets of estimated fish lengths, where the top and bottom lines of each box plot represent the maximum and minimum values, respectively, and the center line connects the MRPE values. The MRPE for the measurements in Group 3 was within 4%, with the error of any single measurement not exceeding 9.5%.

For real-time applications, the proposed method achieves a 35ms inference time when tested on an NVIDIA GTX 1060 GPU. This performance is highly suitable for real-time processing, enabling the system to process 28 frames per second (FPS). This makes the method viable for use in dynamic aquaculture monitoring systems, where real-time feedback is crucial. The fast inference time ensures that the method can be deployed in real-time scenarios, such as live monitoring of fish size and growth in aquaculture environments, as summarized in Table 4.

**Table 4:** Real-time performance on NVIDIA GTX 1060 GPU.

Metric	Value
Inference Time	35 ms
Frames per Second (FPS)	28 FPS
Hardware Configuration	NVIDIA GTX 1060 GPU

To further validate the effectiveness of the proposed method, we performed a t-test to compare the MRPE values of our method (Group 3) with those of existing techniques reported in the literature. Liu et al.^12^ reported an MRPE of 5.5% for keypoint detection and length estimation using a traditional camera system without refraction correction. Yang et al.^13^ achieved an MRPE of 4.2% with a similar setup, but used a more complex multi-camera system. The results of the t-test (p < 0.01) indicated that our method, which incorporates both refraction correction and underwater calibration, significantly outperforms existing methods in terms of accuracy. These statistical results confirm the superior performance of our approach in reducing measurement errors.

We also applied ANOVA to assess the effect of different environmental conditions (e.g., water turbidity and lighting) on measurement accuracy. The ANOVA results showed no significant change in performance across the tested conditions (p = 0.08), indicating that our method is robust and effective under varying underwater conditions.

These findings suggest that our proposed method not only improves the accuracy of fish length measurement but also demonstrates superior performance over existing techniques, offering a reliable and cost-effective solution for real-time underwater measurements in aquaculture environments.

## Discussion

This study introduces a novel method for underwater phenotype measurement of Plectropomus leopardus using deep learning and binocular vision. The method effectively overcomes the challenges associated with keypoint detection and length estimation in aquatic environments. We developed a length measurement algorithm tailored to underwater conditions, which incorporates camera calibration, image distortion correction, and a light refraction model that accounts for secondary refraction. These innovations significantly enhance the accuracy of image transformation. The integration of these advanced measurement techniques not only improves efficiency and accuracy but also reduces labor costs and minimizes the harm to fish associated with traditional contact-based methods.

Despite these promising results, the method has some limitations that need to be addressed in future research. First, while the system was tested and validated under controlled aquaculture conditions with moderate turbidity, its performance under extreme environmental variations-such as high turbidity or fluctuating lighting conditions-remains to be explored. The accuracy of the system in less controlled environments, such as those with high fish density or murky water, is still uncertain. Addressing this issue would ensure the robustness and reliability of the method in real-world aquaculture scenarios.

Additionally, the dataset was specifically designed for Plectropomus leopardus, limiting the method’s applicability to other fish species without additional data and retraining. The model’s ability to generalize across different species is constrained by the species-specific anatomical features and keypoints on which it was trained. Expanding the dataset to include a broader range of species and adapting the model accordingly would enhance its flexibility and generalizability, allowing it to be applied to a wider variety of aquaculture species.

The current implementation also assumes relatively stable fish postures during measurement, which may not always be the case in dynamic or high-density farming environments. Fish in such settings may exhibit rapid or irregular movements, posing challenges for accurate keypoint detection and length measurement. Future research should focus on improving the system’s real-time adaptability to accommodate rapid fish movements and the challenges of high-density environments, which are common in commercial aquaculture operations.

While the refraction correction method significantly improves measurement accuracy, it assumes a relatively stable underwater environment and consistent geometry of the waterproof enclosure. Variations in water quality, enclosure positioning, and camera calibration could still introduce minor errors in the measurements. Further refinement of the refraction model to account for more complex environmental conditions would help improve the precision of the system.

As we continue to refine these technologies, addressing these challenges will be crucial for broader application. Enhancing the system’s robustness under variable environmental conditions, extending the method to include more species, improving real-time adaptability to fish movement, and further refining the refraction correction model will be key steps toward achieving a universally applicable solution for underwater phenotype measurement. Ultimately, these advancements will contribute to the development of smarter, more sustainable food production systems in aquaculture.

## Conclusion

This study introduces a novel, non-invasive method for measuring the phenotype of Plectropomus Leopardus in underwater environments using deep learning and binocular vision. By addressing challenges related to underwater image distortion caused by refraction, we developed a robust length measurement algorithm that integrates camera calibration, distortion correction, and a secondary light refraction model. These innovations ensure accurate image transformation, enabling millimeter-level precision in length estimation.

The results demonstrate the effectiveness of our approach, with the keypoints detection model achieving an accuracy of 98.6% and the fish length estimation method yielding an average measurement error of 3.2 mm. These findings validate the system’s ability to provide precise, contactless measurements in real-world aquaculture environments. Moreover, the method is designed for practical application, leveraging low-cost hardware to ensure affordability and accessibility for widespread use in the aquaculture industry.

While the current study focuses on Plectropomus Leopardus, the methodology has the potential to be adapted to other species with appropriate keypoints definitions and datasets. Future research will aim to enhance the system’s adaptability to varying environmental conditions, such as high turbidity and dynamic fish movements, as well as expand its applicability to a broader range of aquaculture scenarios.

This work represents a significant step toward the intelligentization of aquaculture practices, offering an efficient and sustainable solution for fish phenotype measurement and contributing to the advancement of smart food production systems.

## Related work

One of the simplest methods for measuring fish length is to treat the fish as a rigid body, determining the measurement line as a straight line between its head and tail^14^. However, this approach is less effective when fish bodies are curved in natural environments. A more accurate solution involves extracting a curve along the fish’s longitudinal centerline. This curve can be formed by connecting the midpoints of horizontal lines in the fish image^15^, but approximating the curve with a polyline often introduces significant measurement errors. To improve accuracy, techniques such as third-order regression curves of the fish body centerline^16^ or recursive morphological erosion processes^17^ can be employed. The morphological medial axis is defined as the line connecting all contour points equidistant from both sides. Generating medial axis points begins by determining two endpoints on the contour, followed by recursive morphological erosion to identify candidate points, which are then filtered and connected to form the fish body’s medial axis. Additionally, a distance transform can be applied to the fish contour, where zero values represent the contour and non-zero values represent the background. This method calculates the Euclidean distance from each zero pixel to the nearest non-zero pixel, allowing the ridge line from head to tail to be selected as the measurement line. Morphological thinning and skeletonization methods have also been proposed for length determination^18,19^.

Once the measurement line is established, converting this two-dimensional image information into real-world measurements is necessary. Visual measurement can be achieved using monocular or binocular systems. Monocular estimation requires prior knowledge of the camera’s distance from the fish^20^ or a reference length^21^, which may involve the fish passing through a specific area in the camera’s field of view. In contrast, three-dimensional measurements using binocular cameras are non-intrusive and increasingly precise, with more researchers adopting this method. Harvey^22^ developed an automatic binocular vision measurement system for tuna body length in underwater environments, using farmed tuna approximately 1000 mm long. The results demonstrated measurement errors within acceptable limits, offering valuable insights for advancing automatic fish length measurement.

High-sensitivity standard cameras housed in flat glass waterproof enclosures offer a simple and cost-effective solution for underwater photography. However, during imaging, light refraction occurs as it transitions from water to air, causing the camera’s imaging model to shift from linear to nonlinear. This results in magnification and distortion of underwater targets, hindering the effectiveness of existing stereo vision systems for extracting three-dimensional information^23^. In response, researchers have proposed various solutions to address this refraction issue^24^. Schenker^25^ introduced a method using specially shaped optical components to minimize refraction, though this approach poses implementation challenges due to strict fabrication requirements. Chang^26^ utilized a multi-camera setup to capture images from a single refraction plane, modeling distortion with a depth-dependent approach, but this method also presents complexities due to additional conditions. Treibitz^14^ analyzed refraction distortion through simulations, while Gedge^17^ employed epipolar curves to create a matching cost volume for refraction compensation, though this process is complex and limited in application. Lu^23^ developed a technique for extracting 3D information from underwater circular targets using a monocular camera, but it is restricted to circular shapes. Zhang^27^ proposed a transformation model that converts underwater images to equivalent air images; while this method is easy to implement and broadly applicable, it introduces conversion errors by oversimplifying the model and neglecting secondary refraction at the waterproof enclosure.

As we delve into the transformative impact of advanced computing technologies on fisheries management, it is essential to examine the specific challenges practitioners encounter in the field. One significant challenge is the need for accurate and practical methods to measure fish body length, which is crucial for monitoring fish health and population dynamics. While innovations are emerging, they must balance the demands for precision with the realities of aquaculture environments. This brings us to two important aspects: the applicability of accurate fish body length measurement and the limitations of non-underwater imaging techniques.

1. **Applicability of accurate fish body length measurement:** While studies achieving high precision in measuring fish body length are typically conducted in controlled laboratory settings, these conditions may not reflect the accuracy needed in practical aquaculture scenarios. In contrast, methods designed for real-world environments may be more practical but often lack the necessary measurement precision. Thus, developing an automatic fish body length measurement system that balances high precision with practicality in actual aquaculture settings remains an urgent research challenge.
2. **Limitations of non-underwater imaging:** Many existing fish length estimation methods rely on non-underwater imaging, requiring the removal of fish from their aquaculture environments. This can cause physical harm and physiological stress to the fish, and the sampled individuals may not accurately represent the growth rates of the entire population. Additionally, these methods often necessitate fixing the camera’s distance from the fish or using standard objects as references to establish regression models correlating fish image features with actual length. Given the many uncontrollable variables in real-world aquaculture, non-underwater imaging poses significant challenges for practical fish length estimation.

In this work, we address the conversion errors in previous approaches that fail to account for secondary light refraction. Building on the insights from^27^, we propose a novel underwater light refraction model that includes the effects of secondary refraction. By establishing a precise pixel mapping relationship between underwater images and their corresponding equivalent air images, we introduce an advanced image conversion algorithm. This algorithm calculates the spatial coordinates of each pixel in the underwater image relative to its position in the equivalent air image, enabling accurate transformation of underwater imagery. Our proposed algorithm not only corrects the conversion errors caused by neglecting secondary refraction but also significantly improves the efficacy of underwater image conversion. This enhancement is crucial for optimizing subsequent stages of image processing, ensuring more reliable and accurate analysis of underwater imagery.The comparison of fish length measurement methods in table 5.

**Table 5:** Comparison of fish length measurement methods.

Method	Accuracy (MRPE)	Processing Speed (FPS)	Cost
Liu et al. (2020)^12^	5.5%	30	High (Traditional camera system)
Yang et al. (2021)^13^	4.2%	30	High (Multi-camera system)
Proposed Method (Group 3)	**3.5%**	28	Low (Single calibrated binocular camera)

## Methods

This paper focuses on the Plectropomus Leopardus as the subject of study and employs a combination of deep learning and binocular vision techniques to investigate the identification of species and the estimation of body length for free-swimming fish in an underwater environment, utilizing underwater binocular vision systems. The primary objectives and content of this research are outlined as follows:

1. Employs underwater imagery for the identification of freshwater fish species and the extraction of keypoints information. After evaluating various target detection algorithms, we have chosen the RTMPose network, which is based on a top-down approach, as our detection model due to its high accuracy in detection. The RTMPose network is designed to concurrently identify underwater Plectropomus Leopardus species and detect keypoints information. We collected a series of underwater images featuring 13 Plectropomus Leopardus individuals using a binocular camera system. These images were meticulously annotated to construct a dedicated image dataset for Plectropomus Leopardus. Following dataset augmentation to enhance its diversity and robustness, we trained the RTMPose network. The trained network was then applied to the detection of keypoints information in the underwater images of the 13 Plectropomus Leopardus, thereby validating the model’s detection accuracy.
2. Following the calibration of the underwater binocular camera system, we analyzed the influence of image distortion on the binocular vision measurement algorithm by measuring the standard dimensions of a calibration board. Based on this analysis, we refined the measurement algorithm. By integrating the binocular vision measurement algorithm with the identification of freshwater fish species and the detection of keypoints information, we developed a novel method for estimating the body length of freshwater fish underwater. This method was rigorously tested and validated through a series of experimental evaluations.

### Keypoints detection

Keypoints detection, also known as pose estimation, is a significant research topic in the field of machine vision. With the rapid development of artificial intelligence, keypoints detection based on deep learning has been widely applied in many domains. Its detection effectiveness has qualitatively surpassed that of traditional object detection methods.

#### Model selection

Keypoints detection based on deep learning can be mainly categorized into two types. One type is the region-based top-down (Top-Down) approach, with representative networks such as Mask R-CNN, HRNet, etc. The other type is the regression-based bottom-up approach (Bottom-Up), with representative networks including OpenPose, Part Affinity Fields (PAFs), etc. Compared to the regression-based bottom-up method, the region-based top-down method usually performs better in terms of accuracy but may require higher computational costs because it necessitates object detection first. The bottom-up method may have an advantage in speed, especially when dealing with multi-person scenes, but may face challenges in keypoints grouping, particularly when there is occlusion or interaction among keypoints.

**Fig. 2:**
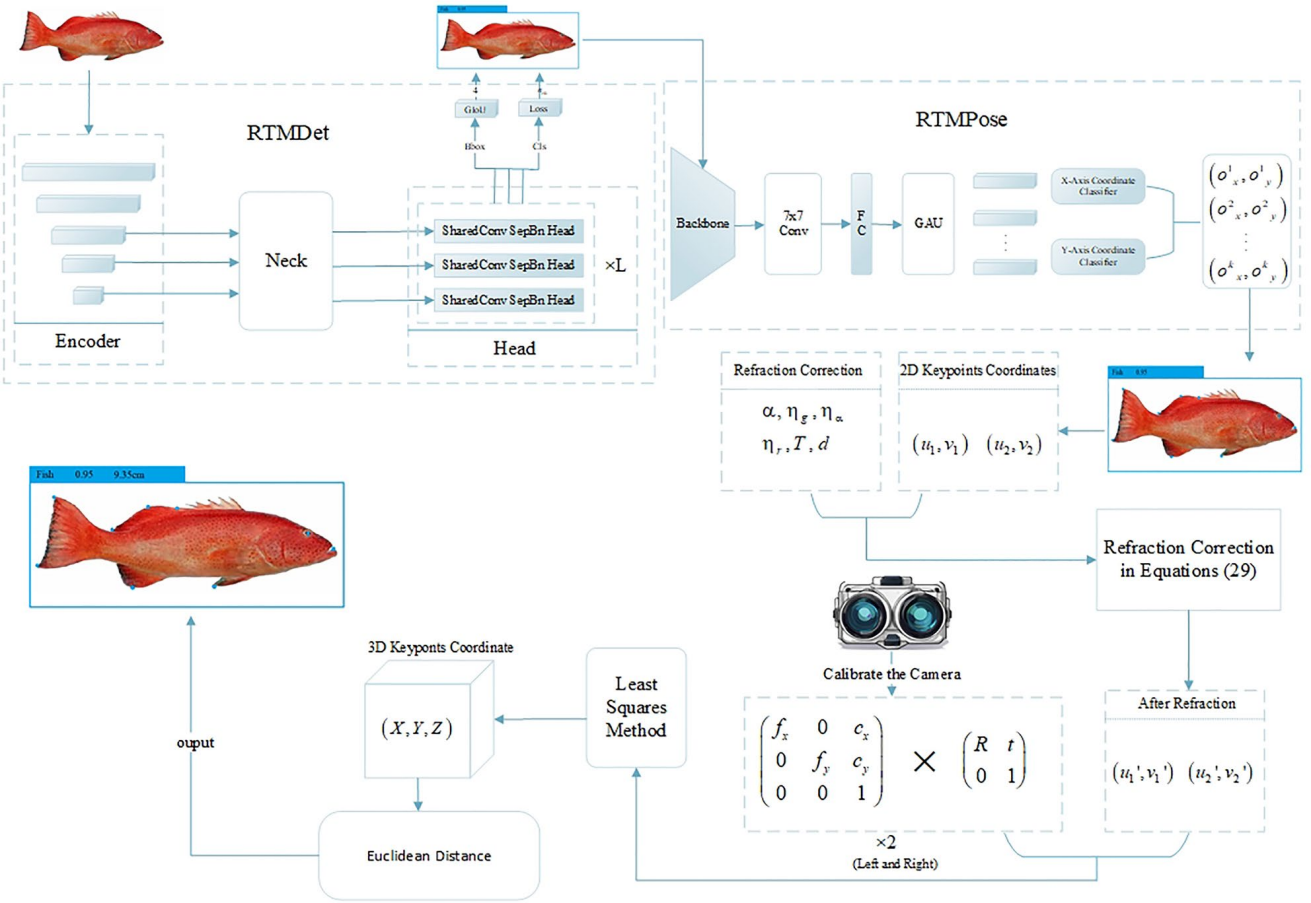
Main workflow.

The Top-Down Approach for keypoints detection involves two stages: the first stage outputs bounding boxes through object detection, and the second stage performs keypoints localization within the bounding box. Figure 2 shows the over-all structure of the framework. The advantage of this approach lies in its ability to improve keypoints localization accuracy through a multi-stage process, as object detection helps the model focus on the regions of interest, thereby reducing the interference from background noise. Since the task of this chapter focuses on the detection of Plectropomus Leopardus, mainly for species identification and keypoints information detection, which demands higher detection accuracy, a Top-Down RTMPose network, used for human pose estimation, is constructed as the method for underwater Plectropomus Leopardus species identification and keypoints information detection.

For effective keypoints detection, the RTMPose network utilizes advanced feature extraction techniques. Specifically, the backbone network in RTMPose is pre-trained using a heatmap-based method^15^ to learn hierarchical features that are critical for detecting keypoints. The model extracts features from the input images through several convolutional layers, followed by feature refinement stages. These features are then used to predict the locations of keypoints. In addition, the UDP method^20^ for backbone pre-training enhances the model’s ability to learn fine-grained features, improving the average precision (AP) from 69.7% to 70.3%. Furthermore, the use of the Exponential Moving Average (EMA) during training helps in mitigating overfitting and stabilizing feature learning, further improving detection accuracy.

#### Dataset establishment

**Fig. 3:**
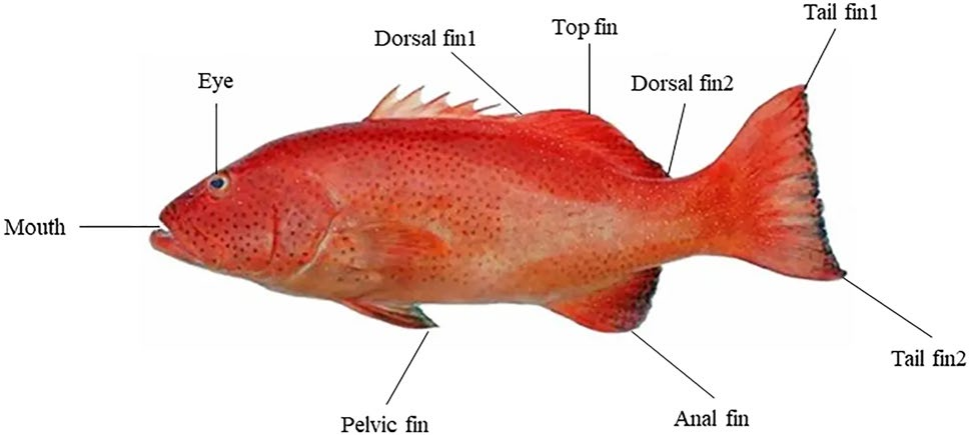
Keypoints definition. Nine keypoints are selected, including mouth, eye, anterior end of dorsal fin (dorsal fin1), posterior end of dorsal fin (dorsal fin2), top of tail fin(tail fin1), bottom of tail fin(tail fin2), anal fin, top fin, and pelvic fin.

As illustrated in Fig. 3^28^, the majority of farmed fish exhibit nine distinct anatomical landmarks where keypoints can be accurately identified. These keypoints include the mouth, eye, the anterior end of the dorsal fin (denoted as dorsal fin 1), the posterior end of the dorsal fin (denoted as dorsal fin 2), the upper portion of the tail fin (referred to as tail fin 1), the lower portion of the tail fin (referred to as tail fin 2), the anal fin, the top fin, and the pelvic fin. The body parts corresponding to these nine keypoints are prevalent across nearly all fish species. Collectively, these keypoints encapsulate the entirety of the fish’s morphology, offering a comprehensive representation of the fish’s posture while swimming. Moreover, this method proves advantageous for improving the accuracy of subsequent reprojection calculations, thereby facilitating more precise analyses in aquatic biomechanics and fish behavior studies.

For this study, we constructed a dataset consisting of 400 images, all sourced from the Deep-sea Innovation Platform in Hainan. To ensure robustness and adaptability, the dataset includes fish of various sizes, postures, and lighting conditions. Initially, the coordinates of the detection frame were output by the object detection module of the RTMDet network. Subsequently, we cropped the images based on these coordinates to obtain a single image of the fish body. We then utilized the open-source image annotation tool *labelme* to annotate the keypoints of the fish, resulting in a dataset of 400 images that include information about the fish body keypoints.

Utilizing the COCO format for annotation, each image incorporated annotations of keypoints, bounding boxes, and binary visibility states, with 1 indicating visible keypoints and 0 for invisible keypoints. Each image consists of 9 keypoints, each presented in the form of (x, y) coordinate and visibility, summarized in a 3x9 matrix. Annotations of images are stored in a JSON file, where they are mapped to their corresponding images. Data was subsequently split into training and test sets, with 69 images used for testing the model and the remaining images used for training purposes.

While the present study focuses on Plectropomus Leopardus, the methodology is adaptable to other fish species. The choice of Plectropomus Leopardus is driven by its significant economic value in aquaculture, making it an ideal subject for this study. However, different fish species exhibit varied anatomical structures, necessitating adjustments to the definitions of keypoints. Extending the current method to other species represents a promising avenue for future research. This would involve recalibrating keypoints and refining the system to accommodate the unique characteristics of each species. Such adaptations would enable the application of the approach to a broader range of aquaculture scenarios and enhance its generalizability across species.

To better understand the model’s robustness, we recognize that further testing on images with different turbidity levels is needed. While our current dataset is limited in terms of highly turbid images, we plan to perform additional experiments to evaluate how varying water clarity influences detection accuracy. This will allow us to refine the model for broader applicability in real-world aquaculture environments.

#### Training techniques

Previous works^15^ demonstrate that pre-training the backbone with a heatmap-based method can enhance model accuracy. We utilize the UDP method^20^ for backbone pre-training, which boosts the model’s average precision (AP) from 69.7% to 70.3%. We employ this technique as a default setting in the subsequent sections.

We adopt the optimization strategy from^29^. The Exponential Moving Average (EMA) is employed to mitigate overfitting, improving the model’s AP from 70.3% to 70.4%. The Flat Cosine Annealing strategy further enhances the accuracy to 70.7% AP. Additionally, we inhibit weight decay on normalization layers and biases.

Following the training strategy in^29^, we employ a strong-then-weak two-stage augmentation approach. Initially, we use stronger data augmentations for 180 epochs, followed by a weaker strategy for an additional 30 epochs. During the strong augmentation stage, we utilize a larger random scaling range of [0.6, 1.4], a larger random rotation factor of 80, and set the Cutout^19^ probability to 1. According to AID^20^, Cutout aids in preventing the model from overfitting to image textures and promotes learning of the pose structure information. In the weaker strategy stage, we disable random shift, use a smaller random rotation range, and set the Cutout probability to 0.5, allowing the model to fine-tune in a domain that more closely aligns with the real image distribution.

### Binocular stereo vision model

**Fig. 4:**
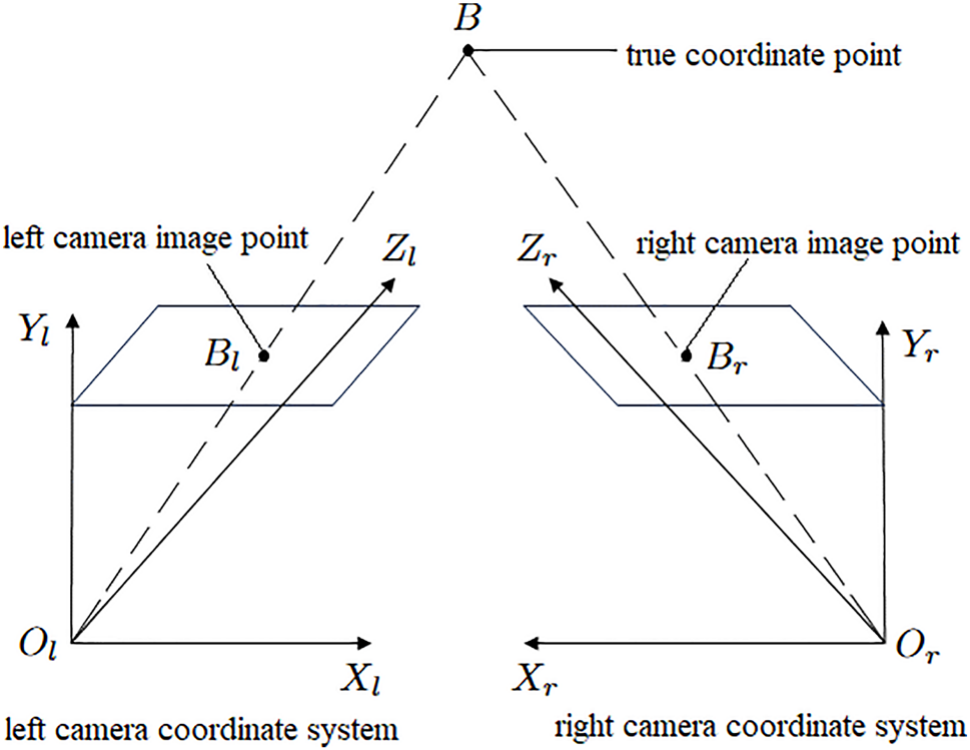
Binocular stereo vision model.

Stereo binocular vision involves capturing an object from different angles simultaneously with two cameras, from which the three-dimensional geometric shape of the object is recovered from two images. As shown in Fig. 4, this is the model of stereo binocular vision.

Assuming $B$ is an arbitrary point in space, $B_l$ and $B_r$ are the projection points of point $B$ onto the left and right images, respectively, and the internal and external parameters of the binocular camera are known, then according to Fig. 4, the relationship between the spatial point $B$ and the points $B_l$ and $B_r$ in the left and right images is given by Eqs. (4) and (5):

4 $$\begin{aligned}&Z_{c1}\begin{bmatrix}u_1\\ v_1\\ 1\end{bmatrix}=\begin{bmatrix}m^1_{11}& m^1_{12}& m^1_{13}& m^1_{14}\\ m^1_{21}& m^1_{22}& m^1_{23}& m^1_{24}\\ m^1_{31}& m^1_{32}& m^1_{33}& m^1_{34}\end{bmatrix}\begin{bmatrix}X\\ Y\\ Z\\ 1\end{bmatrix} \end{aligned}$$

5 $$\begin{aligned}&Z_{c2}\begin{bmatrix}u_2\\ v_2\\ 1\end{bmatrix}=\begin{bmatrix}m_{11}^2& m_{12}^2& m_{13}^2& m_{14}^2\\ m_{21}^2& m_{22}^2& m_{23}^2& m_{24}^2\\ m_{31}^2& m_{32}^2& m_{33}^2& m_{34}^2\end{bmatrix}\begin{bmatrix}X\\ Y\\ Z\\ 1\end{bmatrix} \end{aligned}$$

where $(u_1, v_1, 1)$ and $(u_2, v_2, 1)$ are the homogeneous coordinates of points $B_l$ and $B_r$ in the pixel coordinate system, respectively, and $m_{ij}^{k}$ (with $k = 1,2$; $i = 1, \ldots , 3$; $j = 1, \ldots , 4$) are the elements of the projection matrix $\mathbf {M_k}$ of the left and right cameras at the $i$-th row and $j$-th column, respectively, and $(X, Y, Z, 1)$ are the homogeneous coordinates of the spatial point $P$ in the world coordinate system. By eliminating $Z_{c1}$ and $Z_{c2}$ from the above two equations, we can obtain two sets of linear equations about $X, Y,$ and $Z$:

6 $$\begin{aligned}&{\left\{ \begin{array}{ll} (u_1m_{31}^1 - m_{11}^1)X + (u_1m_{32}^1 - m_{12}^1)Y + (u_1m_{33}^1 - m_{13}^1)Z = m_{14}^1 - u_1m_{34}^1\\ (v_1m_{31}^1 - m_{21}^1)X + (v_1m_{32}^1 - m_{22}^1)Y + (v_1m_{33}^1 - m_{23}^1)Z = m_{24}^1 - v_1m_{34}^1\end{array}\right. } \end{aligned}$$

7 $$\begin{aligned}&{\left\{ \begin{array}{ll} (u_2m_{31}^2-m_{11}^2)X+(u_2m_{32}^2-m_{12}^2)Y+(u_2m_{33}^2-m_{13}^2)Z=m_{14}^2-u_2m_{34}^2\\ (v_2m_{31}^2-m_{21}^2)X+(v_2m_{32}^2-m_{22}^2)Y+(v_2m_{33}^2-m_{23}^2)Z=m_{24}^2-v_2m_{34}^2\end{array}\right. } \end{aligned}$$

The geometric significance of the above two sets of linear equations is that they represent the lines through $O_lB$ and $O_rB$, respectively. Since the spatial point $P$ satisfies Eqs. (6) and (7), the coordinates of the spatial point $B$ (i.e., $X, Y,$ and $Z$) can be solved using the method of least squares.

Through the constraints imposed by the above equations, we take the keypoints coordinates $B_l(u_1, v_1)$ detected in the left image and the keypoints coordinates $B_r(u_2, v_2)$ detected in the right image as input. The least squares method is then used to obtain the actual spatial point coordinates *B*(*X*, *Y*, *Z*). The head keypoints coordinates $h(u_h, v_h)$ and the tail keypoints coordinates $t(u_t, v_t)$ are similarly converted into 3D coordinates $H(X_H, Y_H, Z_H)$ and $T(X_T, Y_T, Z_T)$. The actual body length of the fish can be accurately calculated using the following Euclidean distance equation:

8 $$\begin{aligned} \text {L} = \sqrt{(X_H - X_T)^2 + (Y_H - Y_T)^2 + (Z_H - Z_T)^2} \end{aligned}$$

### Camera calibration

**Fig. 5:**
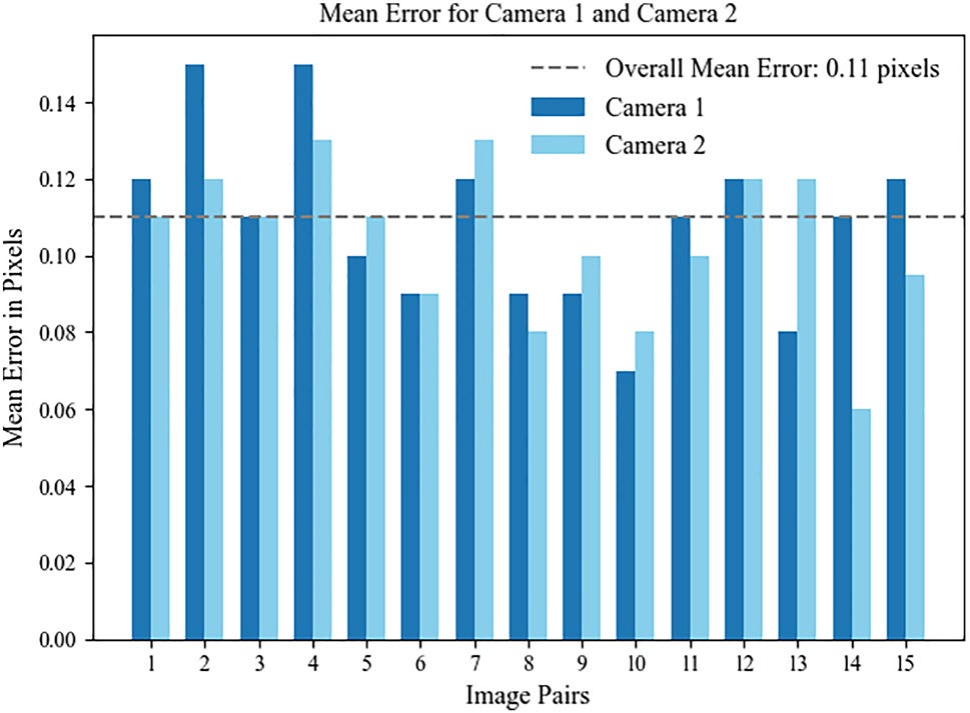
The error caused by the parameters obtained from calibration.

Camera calibration is an important step for binocular vision-based depth estimation, which determines whether the machine vision system can effectively identify, locate and calculate the depth of the object. The Zhang’s calibration method^30^ is adopted, the checkerboard image taken by the camera is used as the reference object, and the coordination relationship between the three-dimensional world to the imaging plane is established through digital image processing and spatial arithmetic operations, then the internal parameter matrix and external parameter matrix of the camera are obtained to perform distortion correction for the collected image. The Fig. 5 shows the error results of the camera calibration.

**Fig. 6:**
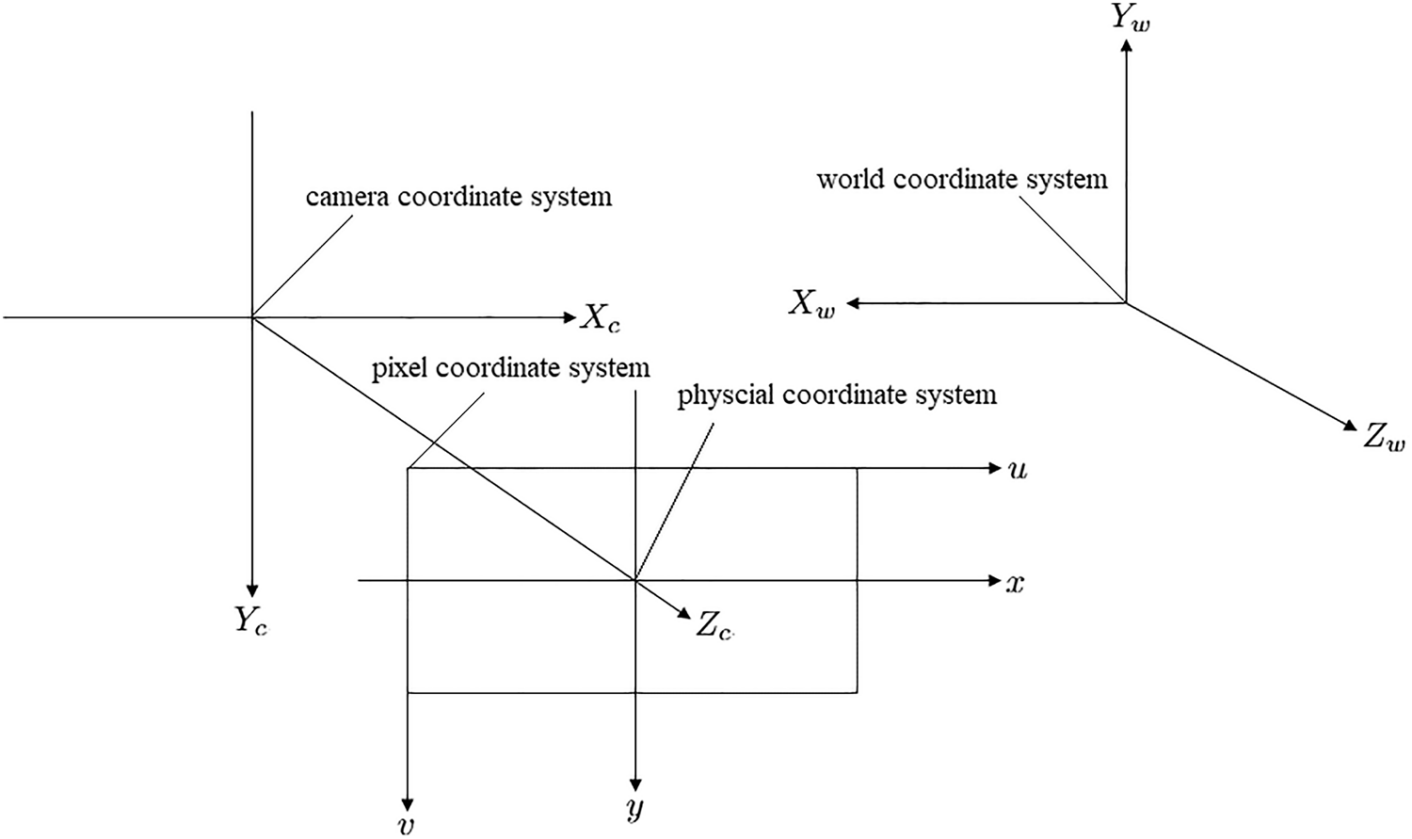
Relationship between coordinate systems.

The world coordinate system is $(X_{W},Y_{W},Z_{W})$, the camera coordinate system is $(X_{C},Y_{C},Z_{C})$ , the image coordinate system is (*x*, *y*) ,and the pixel coordinate system is (*u*, *v*). The mapping among coordinate systems is shown in Fig. 6 and expressed as Eqs. (9)-(11).

9 $$\begin{aligned}&\begin{bmatrix} u\\ v\\ 1\end{bmatrix}=\begin{bmatrix}\frac{1}{dx}& 0& u_0\\ 0& \frac{1}{dy}& v_0\\ 0& 0& 1\end{bmatrix}\begin{bmatrix} x\\ y\\ 1\end{bmatrix} \end{aligned}$$

10 $$\begin{aligned}&z_c \begin{bmatrix} x\\ y\\ 1\end{bmatrix}=\begin{bmatrix} f& 0& 0& 0\\ 0& f& 0& 0\\ 0& 0& 1& 0\end{bmatrix}\begin{bmatrix} x_c\\ y_c\\ z_c\\ 1\end{bmatrix} \end{aligned}$$

11 $$\begin{aligned}&\begin{bmatrix} X_c\\ Y_c\\ Z_c\\ 1\end{bmatrix}=\begin{bmatrix} R& T\\ 0& 1\end{bmatrix}\begin{bmatrix} X_w\\ Y_w\\ Z_w\\ 1\end{bmatrix} \end{aligned}$$

Where *dx* and *dy* represent the proportionality coefficients between image coordinates and pixels, $(u_0, v_0)$ denotes the center pixel coordinate of the image, and *f* is the focal length of the camera. The matrix *R* is a $3 \times 3$ rotation matrix, and *T* is a $3 \times 1$ transformation vector. Based on the aforementioned equations, the mapping between the world coordinate system and the pixel coordinate system is given by Eq. (12)

12 $$\begin{aligned} z_c \begin{bmatrix} u \\ v \\ 1 \end{bmatrix} = \begin{bmatrix} \frac{1}{dx} & 0 & u_0 \\ 0 & \frac{1}{dy} & v_0 \\ 0 & 0 & 1 \end{bmatrix} \begin{bmatrix} f & 0 & 0 & 0 \\ 0 & f & 0 & 0 \\ 0 & 0 & 1 & 0 \end{bmatrix} \begin{bmatrix} {\textbf{R}} & {\textbf{T}} \\ 0 & 1 \end{bmatrix} \begin{bmatrix} X_w \\ Y_w \\ Z_w \\ 1 \end{bmatrix} \end{aligned}$$

The internal and external parameters of the camera can be calculated through the mapping. Assuming that the chessboard is at $Z = 0$ in Zhang’s calibration method, the above formulas can be described as Eqs. (13) and (14).

13 $$\begin{aligned}&s \begin{bmatrix} u \\ v \\ 1 \end{bmatrix} = {\textbf{A}} \begin{bmatrix} {\textbf{r}}_1&{\textbf{r}}_2&{\textbf{r}}_3&{\textbf{t}} \end{bmatrix} \begin{bmatrix} X \\ Y \\ 0 \\ 1 \end{bmatrix} = {\textbf{A}} \begin{bmatrix} {\textbf{r}}_1&{\textbf{r}}_2&{\textbf{t}} \end{bmatrix} \begin{bmatrix} X \\ Y \\ 1 \end{bmatrix} \end{aligned}$$

14 $$\begin{aligned}&{\textbf{A}} = \begin{bmatrix} \frac{f}{d_x} & \gamma & u_0 \\ 0 & \frac{f}{d_y} & v_0 \\ 0 & 0 & 1 \end{bmatrix} = \begin{bmatrix} \alpha & \gamma & u_0 \\ 0 & \beta & v_0 \\ 0 & 0 & 1 \end{bmatrix} \end{aligned}$$

**A** is the camera internal parameter matrix, and *s* is a scale factor.

$H=A[r_1,r_2,t]=[h_1,h_2,h_3]$ is defined as the combination of the internal parameter matrix and the external parameter matrix.*H* is a $3\times 3$ matrix that an element is a homogeneous coordinate, Therefore, there are 8 unknown elements to be solved. Through the formula $\lambda A r[r_1,r_2,t]= [h_1,h_2,h_3]$, where $\lambda$ is a scale factor, introducing the constraint condition $r_1 \times r_2 =0, |r_1|=|r_2|=1$, the solution of the internal parameter can be expressed as Eqs. (15) and (13).

15 $$\begin{aligned}&{\left\{ \begin{array}{ll}r_1=\lambda ^{-1}A^{-1}h_1\\ r_2=\lambda ^{-1}A^{-1}h_2\end{array}\right. } \end{aligned}$$

16 $$\begin{aligned}&{\left\{ \begin{array}{ll}h_1^TA^{-1}A^{-1}h_2=0\\ h_1^TA^{-1}A^{-1}h_1=h_2^TA^{-1}A^{-1}h_2\end{array}\right. } \end{aligned}$$

There are 5 unknowns in *A*. At least 3 different checkerboard pictures are required to solve these unknowns.

### Correction of refraction

In response to the issue of conversion errors introduced by the existing image conversion algorithms due to the neglect of the secondary refraction of light, this paper establishes an underwater light refraction model that considers the secondary refraction of light, and based on this model, derives an underwater image conversion algorithm. This algorithm possesses a more accurate image mapping relationship and can effectively address the problem of conversion errors introduced by the existing image conversion algorithms due to the neglect of the secondary refraction of light.

#### Image conversion algorithm ignoring secondary refraction

**Fig. 7:**
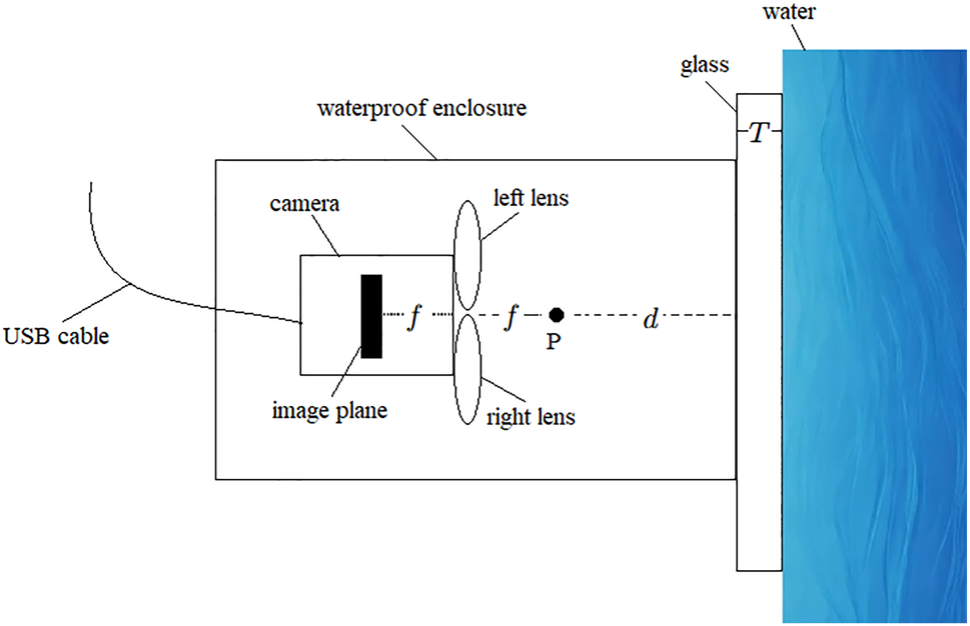
Simplified model of underwater camera.

Underwater cameras typically achieve the function of acquiring underwater images by using a flat shell window to seal a high-sensitivity standard camera in a water-tight manner. This method is simple, economical, and practical. A simplified model of the underwater camera^31^ is shown in Fig. 7.

Where *f* represents the focal length of the camera, *d* represents the distance from the camera’s external focal point to the water-tight enclosure, *T* represents the thickness of the quartz glass of the water-tight enclosure, and *P* represents the camera’s external focal point.

**Fig. 8:**
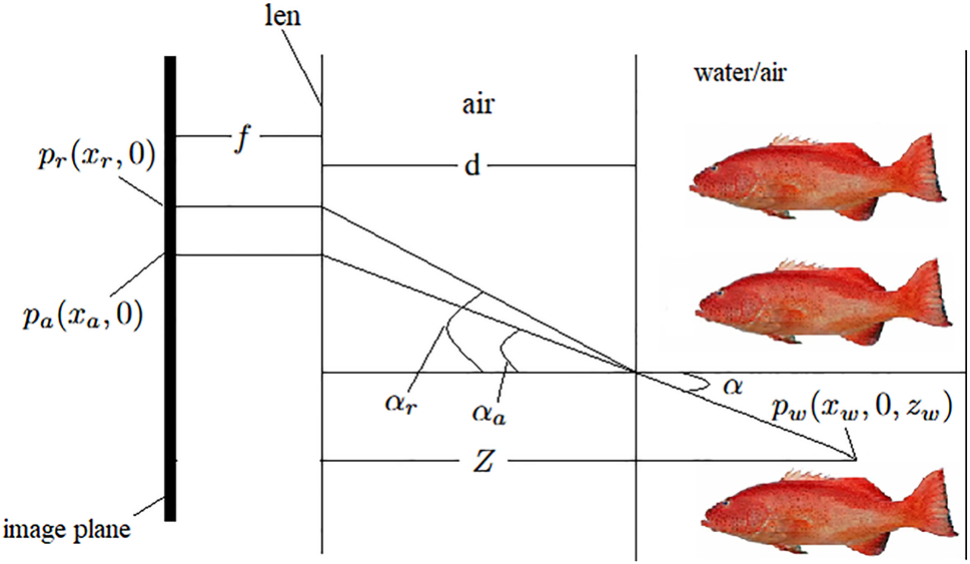
Schematic diagram of the imaging relationship ignoring the secondary refraction of rays.

As can be seen from Fig. 8, light will refract twice due to the change in medium before entering the camera. The first refraction occurs at the interface between water and the quartz glass of the water-tight enclosure, where light passes from water into glass. The second refraction occurs at the interface between the quartz glass of the water-tight enclosure and air, where light passes from glass into air. The refraction of light prevents the stereoscopic vision system in air from being applied in underwater environments. To obtain the equivalent air image from a single underwater image, the air-water interface needs to be set at the camera’s external focal point^30^.

The image conversion algorithm proposed in literature^30^ suggests that, due to the thickness of the water-tight enclosure glass being much smaller than the distance of the target relative to the camera, the propagation process of light in the glass can be neglected. Light only refracts once at the water-air interface before entering the camera. By ignoring the secondary refraction of light, the imaging model is simplified to obtain the mapping relationship between the pixel points of the underwater image and their corresponding air image pixel points.

Without loss of generality, we only discuss the coordinate mapping relationship in one direction. Since the secondary refraction of light is ignored and the interface is located at the focal length, the distance *d* from the camera’s external focal point to the water-tight enclosure and the thickness *T* of the water-tight enclosure glass are zero. The simplified schematic diagram of the imaging relationship is shown in Fig. 8. Where *Z* represents the distance from the target point to the camera, $p_w(x_w, 0, z_w)$ represents the world coordinate system coordinates of the target point, $p_a(x_a, 0)$ represents the camera coordinate system coordinates of the imaging point when the target point is in the air, $p_r(x_r,0)$ represents the camera coordinate system coordinates of the imaging point when the target point is in the water, $\alpha$ represents the angle between the incident light and the normal of the interface, $\alpha _a$ represents the angle between the light and the normal of the interface after passing through the interface when the target is in the air, and $\alpha _r$ represents the angle between the light and the normal of the interface after passing through the interface when the target is in the water.

It can be obtained from Fig. 8:

17 $$\begin{aligned}&{\left\{ \begin{array}{ll} x_r=d\cdot \tan \alpha _r\\ x_w=(Z-d)\cdot \tan \alpha \\ n_a\cdot \sin \alpha _r=n_r\cdot \sin \alpha \end{array}\right. } \end{aligned}$$

Due to the interface being located at the camera’s focal length, the angle between the incident light and the normal of the interface is $\alpha _a$ regardless of whether the target point $p_w(x_w, y_w, z_w)$ is in the air or in the water.

At the same time:

18 $$\begin{aligned} x_a = d \cdot \tan {\alpha _a} \end{aligned}$$

And then we can get:

19 $$\begin{aligned} x_{a}=d\cdot \tan (\arcsin (n_{a}\cdot \sin (\arctan (x_{r}/d))/n_{r})) \end{aligned}$$

It can be concluded from Eq. (18), the pixel points of the underwater image and the air image have a one-to-one correspondence when the interface is located at the camera’s focal length. At this time, under the condition of knowing the camera parameters, only the information contained in an underwater image can be obtained to obtain the equivalent air image. The above algorithm simplifies the imaging model of the underwater camera by ignoring the secondary refraction of light, reducing the complexity of the algorithm. However, in practical applications, the thickness of the waterproof housing of the underwater camera can often reach 5-10 mm, close to the size of the general camera’s focal length. Therefore, in practice, after the light enters the glass, it will travel a long distance in the glass, and at the same time, the refraction occurs at the interface between the glass and the air, if we directly ignore the secondary refraction of light will introduce unnecessary errors, causing the corresponding pixel point position shift, to the subsequent image processing caused by unfavorable effects.

#### Ray refraction model and conversion algorithm based on parallel system

**Fig. 9:**
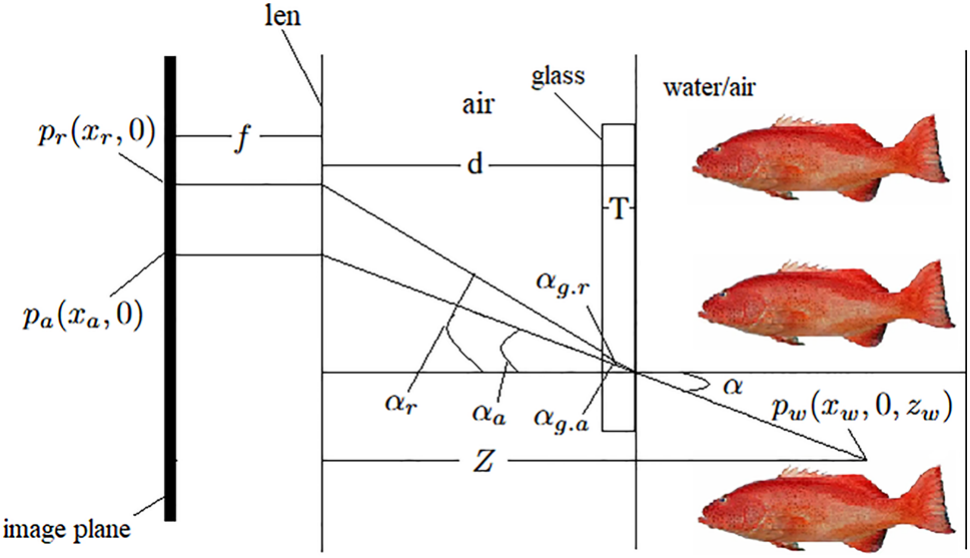
Schematic representation of the imaging relationship based on the parallel system.

The parallel system studied in this paper refers to the underwater camera system where the camera and the water-tight enclosure are placed completely parallel to each other. Consequently, the corresponding schematic diagram of the underwater camera imaging relationship considering the secondary refraction of light can be obtained, as shown in Fig. 9. Without loss of generality, we only discuss the coordinate mapping relationship in one direction.

Where *f* represents the camera’s focal length, *T* represents the thickness of the waterproof enclosure glass, *Z* represents the distance from the target point to the camera, $n_a$, $n_g$, and $n_r$ are the refractive indices of air, glass, and water, respectively, $p_w(x_w, 0, z_w)$ represents the world coordinate system coordinates of the target point, $p_a(x_a, 0)$ represents the camera coordinate system coordinates of the imaging point when the target is in the air, $p_r(x_r, 0)$ represents the camera coordinate system coordinates of the imaging point when the target is in the water, $\alpha$ is the angle between the incident light at the target point and the normal of the interface, $\alpha _{g.a}$ and $\alpha _{g.r}$ are the angles between the light and the normal of the interface after the first refraction when the target is in the air and in the water, respectively, and $\alpha _a$ and $\alpha _r$ are the angles between the light and the glass-air interface normal after the second refraction when the target is in the air and in the water, respectively.

It can be obtained from Fig. 9:

20 $$\begin{aligned}&{\left\{ \begin{array}{ll} x_r=T\cdot \tan \alpha _{g,r}+(d-T)\cdot \tan \alpha _r\\ x_w=(Z-d)\cdot \tan \alpha \\ n_r\cdot \sin \alpha =n_g\cdot \sin \alpha _{g,r}=n_a\cdot \sin \alpha _r\end{array}\right. } \end{aligned}$$

21 $$\begin{aligned}&{\left\{ \begin{array}{ll} x_a=T\cdot \tan \alpha _{g,a}+(d-T)\cdot \tan \alpha _a\\ x_w=(Z-d)\cdot \tan \alpha \\ n_a\cdot \sin \alpha =n_g\cdot \sin \alpha _{g,a}=n_a\cdot \sin \alpha _a\end{array}\right. } \end{aligned}$$

According to Eqs. (20) and (21), the following can be obtained:

22 $$\begin{aligned}&x_r=T\cdot \tan (\arcsin (n_r\cdot \sin \alpha /n_g))+(d-T)\cdot \tan (\arcsin (n_r\cdot \sin \alpha /n_a)) \end{aligned}$$

23 $$\begin{aligned}&x_a=T\cdot \tan (\arcsin (n_a\cdot \sin \alpha /n_g))+(d-T)\cdot \tan \alpha \end{aligned}$$

Among them, the thickness of the waterproof enclosure glass *T*, the camera’s focal length *f*, the refractive index of air $n_a$, the refractive index of water $n_r$, and the refractive index of glass $n_g$ are all known quantities. Therefore, during the image conversion process, the position information of the pixel points in the underwater image can be used to determine the corresponding pixel point position information in the equivalent air image, thereby achieving the conversion from the underwater image to the equivalent air image.

#### Ray refraction model and conversion algorithm based on non-parallel system

**Fig. 10:**
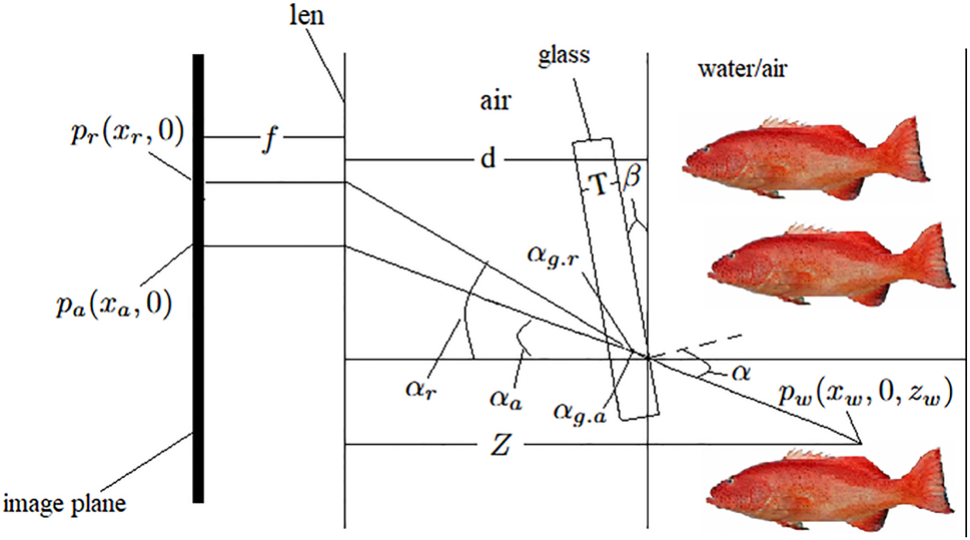
Schematic representation of imaging relationships based on nonparallel systems.

In real-world scenarios, it is challenging for the camera and the waterproof enclosure in an underwater camera system to be placed in a state of complete parallelism. Consequently, a schematic diagram of the underwater camera imaging relationship considering the secondary refraction of light under the non-parallel system state can be obtained, as shown in Fig. 10. Similarly, only the coordinate mapping relationship in one direction is discussed.^32^

Where $\beta$ represents the angle between the plane of the camera placement and the plane of the waterproof enclosure glass.

From Fig. 10, we can obtain:

24 $$\begin{aligned}&{\left\{ \begin{array}{ll} x_r=T\cdot (\tan \alpha _{g,a}-\tan \beta )+(d-\frac{T}{\cos \beta })\cdot \tan (\alpha _\text {r}-\beta )\\ x_w=(Z-d)\cdot \tan (\alpha -\beta )\\ n_r\cdot \sin \alpha =n_g\cdot \sin \alpha _{\text {g.r}}=n_a\cdot \sin \alpha _r \end{array}\right. } \end{aligned}$$

25 $$\begin{aligned}&{\left\{ \begin{array}{ll}x_a=T\cdot (\tan \alpha _{g,a}-\tan \beta )+(d-\frac{T}{\cos \beta })\cdot \tan (\alpha _a-\beta )\\ x_w=(Z-d)\cdot \tan (\alpha -\beta )\\ n_a\cdot \sin \alpha =n_g\cdot \sin \alpha _{g,a}=n_a\cdot \sin \alpha _a\end{array}\right. } \end{aligned}$$

According to Eqs. (24) and (25), the following can be obtained:

26 $$\begin{aligned}&x_r=T\cdot [\tan (\arcsin (n_r\cdot \sin \alpha /n_g))-\tan \beta ]+ \left( d-\frac{T}{\cos \beta }\right) \cdot \tan (\arcsin (n_r\cdot \sin \alpha /n_a)-\beta ) \end{aligned}$$

27 $$\begin{aligned}&x_a=T\cdot [\tan (\arcsin (n_a\cdot \sin \alpha /n_g))-\tan \beta ]+\left( d-\frac{T}{\cos \beta }\right) \cdot \tan (\alpha -\beta ) \end{aligned}$$

In the equation, the angle $\beta$ between the camera and the waterproof enclosure glass, the thickness *T* of the waterproof enclosure glass, and the camera’s focal length *f* can be obtained through measurement, while the refractive index of air $n_a$, the refractive index of water $n_r$, and the refractive index of glass $n_g$ are all known quantities. Therefore, under the non-parallel system, the conversion from the underwater target image to the equivalent air image can also be achieved. In reality, commonly used underwater cameras are designed based on the parallel system. The angle $\beta$ caused by installation errors is a small value. By combining Eq. (26) with Eq. (27), the angle $\beta$ can be neglected, and the commonly used underwater cameras can be treated as a parallel system. Therefore, the subsequent parts of this paper will adopt the image conversion algorithm based on the parallel system for algorithm analysis and experimental comparison.

#### Algorithm analysis of refraction correction

By comparing the image mapping relationships of the existing image conversion algorithms with the image mapping relationships proposed in this paper, we can evaluate the conversion effects of the two algorithms by comparing the distortion of the coordinate points in the two mapping relationships.

In the existing image conversion algorithms, the ratio relationship between $x_r$ and $x_a$ can be derived from Eqs. (18) and (19):

28 $$\begin{aligned} \frac{x_{r}}{x_{a}}=\frac{\tan \alpha _{r}}{\tan \alpha _{a}}=\frac{\tan (\arcsin (n_{r}\cdot \sin \alpha /n_{a}))}{\tan {\alpha }} \end{aligned}$$

In the image conversion algorithm proposed in this paper, the ratio relationship between $x_r$ and $x_a$ can be derived from Eqs. (22) and (23):

29 $$\begin{aligned} T\cdot \tan (\arcsin (n_r\cdot \sin \alpha /n_g))+\frac{x_r}{x_a}=\frac{(d-T)\cdot \tan (\arcsin (n_r\cdot \sin \alpha /n_a))}{T\cdot \tan (\arcsin (n_a\cdot \sin \alpha /n_g))}+ (d - T) \cdot \tan {\alpha } \end{aligned}$$

**Fig. 11:**
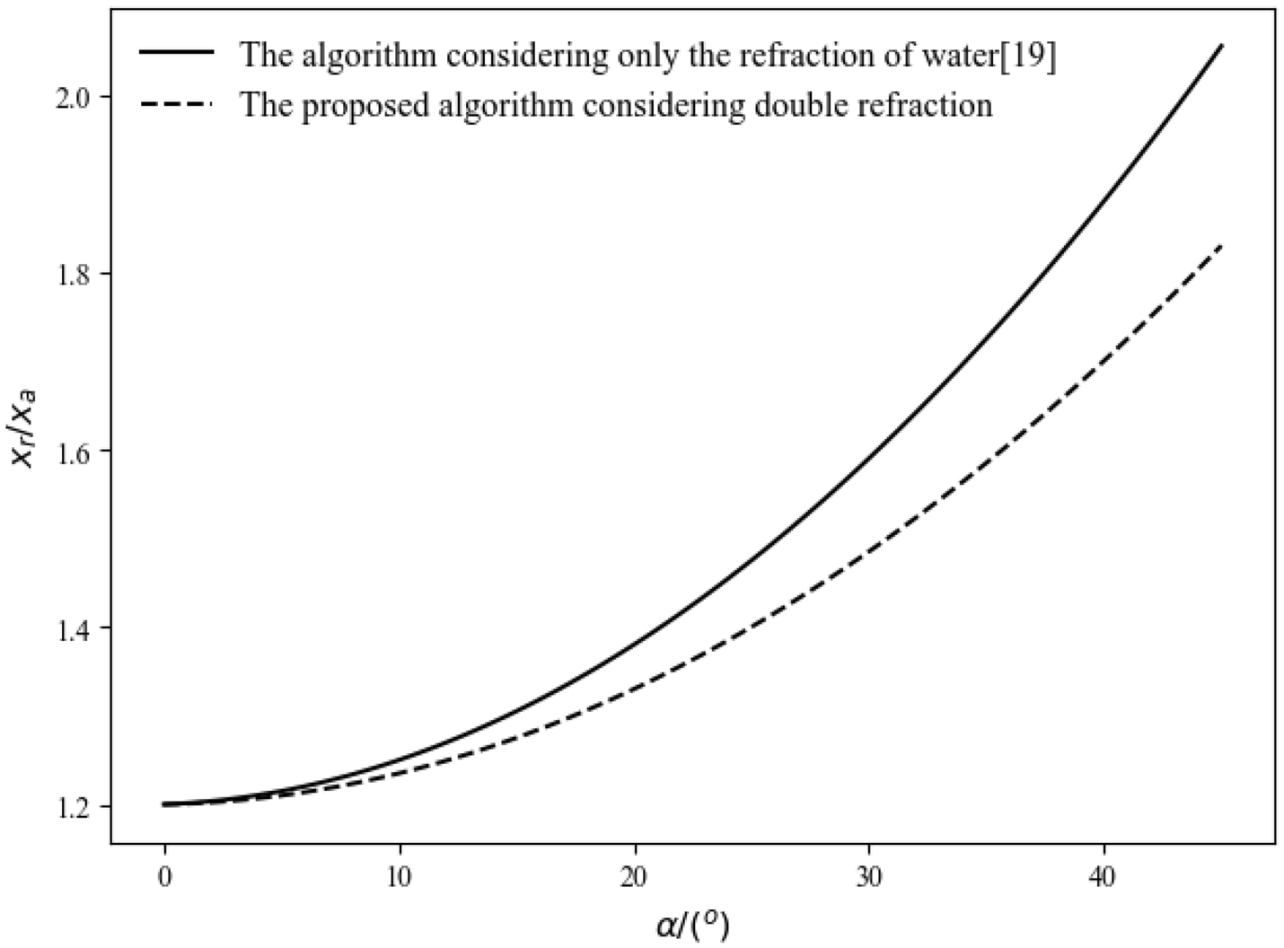
The relationship between $x_r$ and $x_a$ as the incident angle changes.

Setting $n_a = 1$, $n_r = 1.33$, $n_g = 1.6$, $f = 3$ mm, and $T = 2.8$ mm, from Eqs. (25) and (26), we can obtain the relationship between $x_r$ and $x_a$ as the incident angle $\alpha$ changes, as shown in Fig. 11.

As shown in Fig. 11, when the interface is located at the camera’s focal length, refraction causes the image to exhibit pincushion distortion, and the magnification increases with the increase in the incident angle. By comparing the curves of the two algorithms, it can be observed that considering the secondary refraction of light during image conversion can effectively alleviate the pincushion distortion caused by refraction, thus improving the conversion effect of the image conversion algorithm and reducing the positional errors of the pixel points away from the center of the converted image.

## Data Availability

The source code and dataset for this project are available on GitHub. You can access it via the following link: https://github.com/william20001120/measurement-of-fish-length. For cloning the repository, use the following command in your terminal: git clone https://github.com/william20001120/measurement-of-fish-length.git.

## References

[1] Food and Agriculture Organization of the United Nations. The state of world fisheries and aquaculture 2022 (2022).

[2] Group, W. B. The sunken billions revisited: Progress and challenges in global fisheries (2021).

[3] Saberioon, M. & Císař, P. Automated within tank fish mass estimation using infrared reflection system. *Comput. Electron. Agric.* **150**, 484–492 (2018).

[4] Smith, J., Tanaka, A. & Müller, R. Labor cost analysis in modern aquaculture systems. *Aquac. Eng.* **95**, 102214 (2021).

[5] Nguyen, T. L., Wang, Q. & Oppedal, F. Stress-induced mortality in aquaculture: A meta-analysis. *Aquaculture* **528**, 735487 (2020).

[6] Mroz, S. et al. Comparing the quality of human pose estimation with blazepose or openpose. In *2021 4th International Conference on Bio-Engineering for Smart Technologies (BioSMART)*, 1–4 (IEEE, 2021).

[7] Liu, X., Zhang, W. & Li, H. Underwater fish length estimation via deep regression networks. In *Proceedings of the IEEE/CVF Conference on Computer Vision and Pattern Recognition Workshops (CVPRW)*, 2208–2215 (2022).

[8] Thiele, C. J., Hudson, M. D., Russell, A. E., Saluveer, M. & Sidaoui-Haddad, G. Microplastics in fish and fishmeal: an emerging environmental challenge?. *Sci. Rep.* **11**, 2045 (2021). 33479308 10.1038/s41598-021-81499-8 PMC7820289

[9] Lee, H., Park, S. & Kim, M. Multi-interface refraction error analysis in underwater stereo vision. In *Proceedings of the IEEE OCEANS Conference*, 1–6 (IEEE, 2022).

[10] Chen, Y.-R. & Hwang, J.-N. Fish4knowledge dataset. Online Repository (2016).

[11] Hydroacoustic Technology Inc. Eventmeasure ®underwater measurement system specifications. Product Brochure (2023).

[12] Suo, F., Huang, K., Ling, G., Li, Y. & Xiang, J. Fish keypoints detection for ecology monitoring based on underwater visual intelligence. In *2020 16th International Conference on Control, Automation, Robotics and Vision (ICARCV)*, 542–547 (IEEE, 2020).

[13] Voskakis, D., Makris, A. & Papandroulakis, N. Deep learning based fish length estimation. an application for the mediterranean aquaculture. In *OCEANS 2021: San Diego–Porto*, 1–5 (IEEE, 2021).

[14] Zhou, J., Shi, B., Liu, G. & Ju, S. Accuracy analysis of dam deformation monitoring and correction of refraction with robotic total station. *PLoS One* **16**, e0251281 (2021). 33956839 10.1371/journal.pone.0251281 PMC8101930

[15] Li, J. et al. Human pose regression with residual log-likelihood estimation. In *Proceedings of the IEEE/CVF International Conference on Computer Vision*, 11025–11034 (2021).

[16] Miranda, J. M. & Romero, M. A prototype to measure rainbow trout’s length using image processing. *Aquac. Eng.* **76**, 41–49 (2017).

[17] Guan, J. et al. Automated pixel-level pavement distress detection based on stereo vision and deep learning. *Autom. Constr.* **129**, 103788 (2021).

[18] McNally, W., Vats, K., Wong, A. & McPhee, J. Rethinking keypoint representations: Modeling keypoints and poses as objects for multi-person human pose estimation. In *European Conference on Computer Vision*, 37–54 (Springer, 2022).

[19] DeVries, T. & Taylor, G. W. Improved regularization of convolutional neural networks with cutout. arXiv preprint arXiv:1708.04552 (2017).

[20] Huang, J., Zhu, Z., Guo, F. & Huang, G. The devil is in the details: Delving into unbiased data processing for human pose estimation. In *Proceedings of the IEEE/CVF Conference on Computer Vision and Pattern Recognition*, 5700–5709 (2020).

[21] Maji, D., Nagori, S., Mathew, M. & Poddar, D. Yolo-pose: Enhancing yolo for multi person pose estimation using object keypoint similarity loss. In *Proceedings of the IEEE/CVF Conference on Computer Vision and Pattern Recognition*, 2637–2646 (2022).

[22] Furuta, N., Tanaka, T. & Komeyama, K. Method for automating fish-size measurement and camera calibration using a three-dimensional structure and an optical character recognition technique. *Nippon Suisan Gakkaishi* **87**, 100–107 (2021).

[23] Lu, H., Li, Y. & Serikawa, S. Computer vision for ocean observing. *Artif. Intell. Comput.* 1–16 (2017).

[24] Marini, S. et al. Tracking fish abundance by underwater image recognition. *Sci. Rep.* **8**, 13748 (2018). 30213999 10.1038/s41598-018-32089-8 PMC6137190

[25] Garcia, R. et al. Automatic segmentation of fish using deep learning with application to fish size measurement. *ICES J.Mar. Sci.* **77**, 1354–1366 (2020).

[26] Zhang, J., Chen, Z. & Tao, D. Towards high performance human keypoint detection. *Int. J. Comput. Vis.* **129**, 2639–2662 (2021).

[27] Zhang, W., Deng, X., Zhang, Q. & Li, H. Non-parallel system underwater image transformation model. *Acta Photonica Sinica* **44**, 211002 (2015).

[28] Dong, J. et al. A detection-regression based framework for fish keypoints detection. *Intell. Mar. Technol. Syst.* **1**, 9 (2023).

[29] Lyu, C. et al. Rtmdet: An empirical study of designing real-time object detectors. arXiv preprint arXiv:2212.07784 (2022).

[30] Zhang, Z. Flexible camera calibration by viewing a plane from unknown orientations. In *Proceedings of the Seventh IEEE International Conference on Computer Vision*, vol. 1, 666–673 (Ieee, 1999).

[31] Hornberg, A. & GODDING, R. Camera calibration. *Handbook of Machine and Computer Vision* 291–316 (2017).

[32] Shi, C., Wang, Q., He, X., Zhang, X. & Li, D. An automatic method of fish length estimation using underwater stereo system based on labview. *Comput. Electron. Agric.* **173**, 105419 (2020).

